# Striving to protect and reconnect: narratives of motherhood in the context of homelessness

**DOI:** 10.1186/s12939-026-03087-4

**Published:** 2026-10-29

**Authors:** Tommy Carlsson, Elisabet Mattsson, Jenny Eriksson Lundström, Sophie Nadia Gaber, in collaboration with the Women’s Advisory Board for Inclusion Health

**Affiliations:** 1 Department of Women’s and Children’s Health, CIRCLE – Complex Intervention Research in Health and Care, Uppsala University, Uppsala, Sweden; 2 Department of Health Sciences, The Swedish Red Cross University, Huddinge, Sweden; 3 Department of Healthcare Sciences, Marie Cederschiöld University, Stockholm, Sweden; 4 Department of Informatics and Media, Uppsala University, Uppsala, Sweden; 5 Ersta Möjlighet, Stockholm, Sweden

**Keywords:** Homeless, Inclusion health, Patient and public involvement, Qualitative research, Women’s health

## Abstract

**Background:**

Homelessness is a critical determinant of health that disproportionately affects women and children. While adverse health consequences of homelessness are well established, less is known about how mothers sustain care, dignity, and wellbeing under such conditions. Drawing on feminist perspectives and an Inclusion Health framework, the aim was to explore women’s lived experiences of motherhood in the context of homelessness.

**Methods:**

A qualitative study was conducted using narrative interviews with twelve women in Sweden who had lived experience of homelessness and were mothers of children under eighteen. Participants were recruited through four civil society organisations. Data were analysed using Lindseth and Norberg’s phenomenological–hermeneutic method, involving naïve reading, structural analysis, and comprehensive interpretation. A participatory approach was applied through collaboration with the Women’s Advisory Board for Inclusion Health, comprised of women with lived experience of homelessness, who contributed as co-researchers to study design and interpretation of findings.

**Results:**

Through our interpretation of the narratives, motherhood was understood as both a source of vulnerability and a moral practice through which women sustained family life amid systemic adversity. Three interrelated themes characterised the women’s experiences. First, mothers encountered fragmented welfare systems that demanded accountability yet offered limited continuity or trust, compelling them to struggle for recognition and care. Second, housing instability and separation from children fractured the women’s sense of identity and belonging, revealing how structural insecurity and stigma disrupted maternal roles. Third, mothers sought to protect and reconnect with their children, demonstrating resilience as a relational process rooted in care, responsibility, and love.

**Conclusions:**

Motherhood in the context of homelessness is an existential and moral struggle shaped by structural insecurity that constrains mothers’ agency and undermines their credibility. Maternal resilience is revealed not just as personal endurance, but also as a relational process grounded in connection, care, and responsibility. Advancing health equity for mothers experiencing homelessness requires welfare and health systems that prioritise relational trust, continuity of support, and the recognition of lived experience as legitimate knowledge.

**Supplementary Information:**

The online version contains supplementary material available at https://doi.org/10.1186/s12939-026-03087-4.

## Introduction

Women’s experiences of homelessness are shaped by intersecting experiences of violence, poverty, stigma, and structural insecurity [1]. For mothers, homelessness can extend beyond the loss of stable housing to disrupt safety, family relationships, caregiving, and a sense of belonging, with consequences for both maternal and child wellbeing [2, 3]. Motherhood in the context of homelessness therefore needs to be understood not only in relation to individual circumstances, but also to the social and structural conditions that shape women’s opportunities to care for themselves and their children. Drawing on feminist ethics of care, care is understood in this study as a relational and situated practice encompassing the practical, emotional, and social work through which women attend to their own and their children’s needs, sustain relationships, and enact responsibility within the possibilities and constraints of their circumstances [4]. From a health equity perspective, these conditions can create avoidable and unjust differences in opportunities for health and wellbeing [5].

Existing research has largely focused on the risks and outcomes associated with homelessness, reporting high rates of trauma, stress, and child adversity [6]. However, less attention has been paid to women’s own meaning-making, agency, and care practices within these conditions, reflecting a key limitation in current health research [7]. With the majority of the literature on homelessness focusing on men and general research questions, there is a scarcity of exploratory research addressing the complex and specific circumstances of women [8]. Mothers experiencing homelessness are often portrayed as vulnerable recipients of care, rather than as moral agents who sustain family life, assume responsibility, and preserve integrity under structural insecurity. However, much is still unknown about the agency and resilience of mothers with lived experience of homelessness.

The concept of resilience has traditionally been used to describe adaptation to adversity, focusing on personal and proximal protective factors rather than broader structural conditions [9], and later, the daily relational and contextual processes that enable adaptation [10]. More recent work conceptualises resilience as a dynamic, relational, and context-dependent process that is embedded in social connection, community resources, and supportive environments [11, 12]. In the context of homelessness, where structural insecurity shapes daily life, understanding resilience as a socially situated process is particularly critical. While limited studies illustrate strands of positive functioning and resilience among parents experiencing homelessness, the overall lack of studies highlights a gap in the understanding of promotive and protective factors that contribute to resilience over time [13]. Although it is probable that homelessness has a substantive impact on parental mental health, there is a need for exploratory studies providing contextual in-depth insights [14].

Through a feminist and phenomenological–hermeneutic lens, this study places lived experience, moral agency, and interpretation in the foreground. It is also informed by an *Inclusion Health* framework, an equity-oriented model developed to improve health outcomes among socially excluded populations by addressing structural barriers, stigma, and systemic neglect [15, 16]. Within this perspective, *Inclusion Health* extends the principles of health equity into practice, emphasising participation, recognition, and justice. Recent research further highlights the importance of relational practice in health and social services, an approach that prioritises human connection, mutuality, and responsiveness as central to effective care and support [17]. Such perspectives resonate with feminist and phenomenological understandings of ethics as relational, grounded in attentiveness, trust, and moral responsibility rather than procedural rules.

### Aim

The aim was to explore women’s lived experiences of motherhood in the context of homelessness.

## Method

### Study design

This study is part of a broader research programme seeking to promote inclusion health among women with lived experience of homelessness. It was designed as a qualitative study informed by phenomenological–hermeneutical lens [18, 19] and based on narrative interviews exploring women’s lived experience of motherhood in the context of homelessness. The study is reported in accordance with the Consolidated Criteria for Reporting Qualitative Research (COREQ) [20].

Guided by Medina’s [7] concept of epistemic injustice, the study explicitly sought to redress structural silencing by recognising women’s experiential knowledge as legitimate evidence within health and social policy contexts. This theoretical stance informed both the participatory design and the interpretive approach, ensuring that knowledge was co-produced and grounded in participants’ lived experience.

A participatory approach was applied through collaboration with the Women’s Advisory Board for Inclusion Health (WAB), a group of women with lived experience of homelessness. The WAB was established in 2020 as part of the broader research programme and has since employed women with lived experience as co-researchers through regular workshops, providing continuity and trust in the collaboration. Membership of the board has varied over time due to members’ changing social circumstances. At the time this study was planned and conducted, six women were employed in the WAB.

The WAB initiated the idea for this study and was actively involved in its design. The WAB also co-developed the interview guide and created the information materials used for informed consent. In addition, one woman with lived experience of motherhood in the context of homelessness participated in the analysis and interpretation of the data.

### Study setting

This study was conducted in Sweden, in which more than 27,000 adults were identified as experiencing homelessness during 2023. Approximately 32%, around 8,800 individuals, were women, and at least 9,400 children were affected by homelessness [21]. The national estimate is based on administrative data collected according to the National Board of Health and Welfare’s definition of homelessness, which includes adults in acute, institutional, or transitional housing situations. However, the survey excludes several groups, including asylum seekers and undocumented migrants, and was affected by substantial underreporting from municipalities. Consequently, the figures are considered to represent a minimum estimate, and the actual prevalence of homelessness in Sweden, particularly among women in vulnerable life situations, is likely to be higher.

In Sweden, social work is regulated by the Social Services Act (SFS 2025:400), which gives municipalities the main responsibility for providing welfare services. This decentralised system means that municipalities play a central role in supporting families, including those experiencing homelessness, although the range and quality of services may vary locally [22]. Municipal social services have a broad mandate covering economic assistance, child welfare, and support for vulnerable groups, including families experiencing homelessness and individuals exposed to intimate partner violence.

The National Board of Health and Welfare has the overall responsibility for social services, healthcare, and child protection. In 2020, the United Nations’ Convention on the Rights of the Child (CRC) [23] was incorporated into Swedish law [24]. While not all provisions are directly applicable, the incorporation requires that Swedish authorities interpret national law in line with the CRC, with particular emphasis on the best interests of the child (Article 3) and the right to an adequate standard of living (Article 27) [23].

### Participants

We employed both purposive and convenience sampling to recruit women experiencing homelessness, in collaboration with four civil society organisations, three based in Stockholm, Sweden’s largest city (> 1,000,000 inhabitants), and one in Uppsala, Sweden’s fourth largest city (> 240,000 inhabitants). This approach was chosen because these organisations provide direct services to women experiencing homelessness and therefore offered a feasible way to reach this population. Collaboration with multiple organisations in two cities increased the likelihood of variation in participants’ backgrounds and experiences. Inclusion criteria were: (i) adult women aged 20 years or older, (ii) lived experience of homelessness, (iii) at least one biological child under the age of 18, and (iv) able to communicate in Swedish. Lived experience of homelessness was defined in accordance with the four categories of the European Typology of Homelessness and Housing Exclusion (ETHOS) [25].

Women expressing an interest to participate received both written and verbal information about the study from staff of the civil society organisations that assisted with the recruitment. In total, 16 women expressed interest. Of these, one relocated before the interview and three declined due to scheduling conflicts or fatigue, resulting in a final sample of 12 participants (Table 1). The mean age of participants was 38.4 years (range: 26–57), and the average number of children was 2.3 (range: 1–7). Five women had sole custody of their children, three shared custody, one shared custody of her youngest child only, and three had no custody.

**Table 1:** Sociodemographic characteristics of the participants (*n* = 12)

Characteristic		*n*
Highest level of education	University/university college	2
	Upper secondary school	6
	Vocational school	2
	Compulsory school	1
	Did not complete compulsory school	1
Years in homelessness	≤ 1	3
	1–5	4
	6–10	2
	> 10	3
Housing situation	Roofless	1
	Protected housing (due to domestic violence)	3
	Provided by social services or civil society organisations	3
	Social service support and regular monitoring regarding childcare responsibilities	2
	Permanent residence	3

### Data collection

Between February and October 2024, participants were individually interviewed at a location of their choice. Two researchers (EM, JEL) conducted the interviews together, with one acting as interviewer and the other as observer, asking follow-up questions to elicit rich and nuanced narratives [26]. All interviews were audio recorded and transcribed verbatim. A semi-structured narrative-oriented interview guide containing open-ended questions was used, with interviews structured into four phases. In the introductory phase, participants were asked, *“Could you tell us a little about yourself and your background?”* During the narrative phase, they were invited to tell their stories and describe specific events or situations that had influenced their experience of motherhood in the context of homelessness. The follow-up phase included prompts such as, *“Is there anything else you would like to share about your experience of motherhood in the context of homelessness?”* and *“Do you have any personal reflections on your role as a mother and your situation?”* In the closing phase, participants were asked, *“Is there anything else you’d like to add?”* In addition, a short set of background questions was included, covering age, education, housing situation, duration of homelessness, number of children, and custody arrangements. The interview guide was co-developed with women with lived experienced of homelessness from the WAB but was not piloted.

Prompts and follow-up questions were used to encourage storytelling and reflection, in line with the narrative orientation of the interviews. The average interview duration was 38 min (ranging from 17 to 58 min). After each interview, the researchers wrote memos summarising the discussion and noting initial impressions. A brief summary of the interview was then prepared and shared with participants shortly after the interview, prior to the later stages of analysis. Four participants provided feedback. All were satisfied with the summaries and no new information relevant to the analysis was identified. This process was intended to support accurate representation of participants’ experiences.

### Data analysis

Data were analysed using Lindseth and Norberg’s phenomenological-hermeneutical method [18, 19], which builds on Paul Ricoeur’s theory of interpretation [27]. This method has previously been applied to interpret narrative interview data exploring lived experiences of homelessness [28]. Three research team members (i.e., two academic researchers (SNG and TC) and a co-researcher with lived experience of motherhood in the context of homelessness from the WAB) performed the analyses based on the following three stages: naïve reading, structural analysis, and comprehensive understanding. In preparation for the naïve reading stage, the interview audio-recordings were transcribed verbatim by a professional transcription service and pseudonymised to preserve participants’ confidentiality. The transcripts were translated collaboratively from Swedish into English by the four academic researchers, one a native English speaker. Wording and tone were discussed with the co-researcher with lived experience and the WAB, referring back to the Swedish transcripts to preserve the women’s expressions and intended meaning.

During the naïve reading stage, the interview transcripts were read repeatedly to gain an initial understanding of the whole, allowing preliminary interpretations to be developed in dialogue with the text. Together with the broader research team, the three research team members primarily responsible for the analysis discussed their reflections and formulated the naïve understanding. Next, the three research team members independently identified meaning units in the text (i.e., significant excerpts from the interview transcripts), which were subsequently discussed together with the broader research team. The interview memos were also reviewed at this stage to help contextualise the text through their impressions and reflections after each interview. During the structural analysis, the three research team members moved between the parts and the whole, reflecting on the text while identifying and interpreting patterns of meaning and re-reading the naïve understanding to promote coherence. Through an iterative interpretative process, the meaning units were condensed and reformulated based on the interpretations of the three research team members. These condensations were further synthesised into themes that express the essential meaning of motherhood in the context of homelessness. The preliminary themes were discussed and refined together with the broader research team and the WAB whose insights as experts with lived experience of motherhood in the context of homelessness contributed to the interpretive process.

In the findings section, quotations from the transcribed interviews are used to support and illustrate the interpretations. Finally, the themes were reflected upon in relation to relevant literature and theory, and the comprehensive understanding of the findings is presented in the discussion section.

### Ethical considerations

This study was approved by The Swedish Ethical Review Authority (No. 2023-05286-01) and adhered to the principles of the Declaration of Helsinki [29]. Before each interview, the researcher explained the interview process and addressed any questions or concerns raised by participants. Written informed consent was obtained prior to participation. Participation was voluntary and participants had the option to withdraw at any time without providing a reason. Participants received a gift voucher valued at 13 Euros as compensation for their time and contribution.

Members of the WAB were compensated for their time and engagement through employment within the research programme at Marie Cederschiöld University, Stockholm, Sweden, in line with ethical principles of equity and fairness and consistent with the National Institute for Health and Care Research (NIHR) guidelines on public involvement in research [30]. As most WAB members live under protected personal identity, their names are not disclosed in publications. This was a collective decision made together with the board to maintain the women’s safety and confidentiality.

Participatory approaches increasingly include data collection conducted by peer interviewers; however, this was not feasible in the present study. Several interviews took place in protected housing facilities, and it was essential to ensure that the location and address of these facilities remained undisclosed. To protect the safety and anonymity of both participants and WAB members, the interviews were conducted by the academic researchers, while the WAB members contributed to the study’s design, recruitment materials, and interpretation of the findings.

### Rigour and reflexivity

Rigour was enhanced in line with Lincoln and Guba’s [31] four criteria of trustworthiness in qualitative research: credibility, dependability, confirmability, and transferability [31]. Credibility was supported through ongoing critical reflections on the research team’s individual and shared preunderstandings with attention to positionality, interpretive responsibility, and language. This reflexive stance aligned with feminist and phenomenological–hermeneutic traditions, as well as Inclusion Health principles emphasising equity, participation, and relational trust.

Consistent with a phenomenological–hermeneutic approach, member checking was used not as validation but to ensure that interpretations resonated with participants’ lived experiences [32]. This process recognised participants’ role in co-constructing knowledge and enabled clarification during data collection and analysis.

The interdisciplinary research team consisted of members of the WAB and four academic researchers (four women and one genderqueer man). The WAB members, all women with lived experience of homelessness, acted as co-researchers and contributed to study design, informed consent materials, data interpretation, and manuscript review. One of the co-researchers with lived experience has published a narrative drawing on her lived experience of motherhood, homelessness, and gender-based violence [33], providing additional context for the experiential perspectives informing the analysis in the present study. Among the academic researchers, four held doctoral degrees (PhDs) and were associate or full professors with extensive experience in qualitative research. Their professional backgrounds included two specialist nurse-midwives (TC, EM), an occupational therapist (SNG), and a social scientist with expertise in information systems (JEL). The team sought to integrate and value the varied lived and professional experiences represented.

## Findings

### Naïve reading

Through reading the women’s narratives, we developed an initial understanding of motherhood in the context of homelessness as a state of continuous struggle and negotiation. Women described having to detach from their emotions to prioritise their children, while coordinating services failing to provide support. Feeling scrutinised and undermined by authorities was recurrent, evoking a sense of exclusion and betrayal. There was profound sorrow over the inability to provide a safe home and over witnessing intergenerational cycles of violence, abuse, and trauma. Losing custody meant separation and a sense of becoming a replaceable stranger in their children’s lives. However, beneath this suffering was enduring love and responsibility, as the determination to secure safety and wellbeing for their children remained unbroken. Overall, we interpreted motherhood in the context of homelessness as an existence marked by love and loss, resistance and surrender, and the ongoing effort to preserve maternal identity amid structural and relational insecurity. This naïve understanding guided the subsequent structural analysis.

### Structural analysis

Three themes were identified, illuminating different dimensions of the essential meaning of motherhood in the context of homelessness.

#### Theme 1: Mother against Goliath: battling a complex labyrinth of bureaucracy and fragmented services

The first theme illuminates how the lived meaning of motherhood in the context of homelessness unfolded as a struggle within systems of institutional neglect and control. It highlights how navigating a complex bureaucracy and fragmented services became an inseparable part of being a mother in this context. Encounters with multiple agencies demanded relentless self-advocacy, revealing that homelessness was experienced not merely as an absence of shelter but as an ongoing negotiation with institutional power. Fragmented services amplified isolation and instability, perceived to prioritise procedures over empathy. Being a “mother against Goliath” captures the lived tension between agency and powerlessness, between striving to protect one’s children and confronting bureaucratic indifference that constantly questions one’s legitimacy as a mother.

In the face of institutional inaction and inconsistent support, women were forced to take on responsibility of self-advocacy extending far beyond their expected role as mothers, blurring the boundary between caregiving and survival. They described the pressure to independently navigate disconnected systems despite lacking resources or guidance, illustrating how responsibility was displaced from the state onto the individual.

I’m nine months pregnant […] but during these nine months they [social services] didn’t do a damn thing except talk about and say, ‘the supported housing you have now, you can’t live in when your child is born. You get to solve it.’ Well, how should I solve it? I would never have been able to solve my housing situation. (Interview 6)

The pressure extended beyond securing accommodation. One mother, who had experienced repeated moves after separating from her partner and was involved in a custody dispute, described having to manage multiple demands while caring for her child:

During these processes, there is often an almost superhuman amount of pressure from all sides, because it’s not just one thing you have to sort out, but everything. It’s like being bombarded from all sides. So, yes, if you care about the child, you should make sure you care about the parent who takes care of them, I think. (Interview 11)

The lived experience of motherhood in the context of homelessness meant living under constant scrutiny. Feeling judged and dismissed by authorities eroded the women’s trust in authorities and undermined their sense of competence and worth as mothers. Women’s accounts of unempathetic and unaware approaches experienced from authorities and services further increased tensions and mistrust. One mother, who had moved repeatedly during pregnancy and after her child’s birth, lived with her partner and child in an apartment arranged through social services. She described:

I feel very insecure about whether we’re going to have housing support workers and for how long […] it’s not our place, it’s still through the authorities […] I feel watched […] when I’m involved with social services and they want to check this and that […] I want to feel like a parent, and I’ve had an apartment before […] it feels like they’re treating me as if I’m incapable in some way. And that’s not how I want to feel. (Interview 5)

Her words illustrate how institutional oversight reinforced the experience of being under constant surveillance, blurring the boundary between support and control. At times, women described being pushed to the brink in order to access basic support:

I call the social services […] they told me […] ‘We can’t do anything until you stand on the street with the children.’ But are you kidding me, I said. I didn’t want to stand on the street with the children, I want to plan ahead. (Interview 1)

In these narratives, seeking help was understood as both an act of courage and a source of humiliation. Mothers described a paradoxical experience of dependence and resistance, having to fight the very systems meant to protect them. They felt compelled to assume the role of coordinators between fragmented services, often repeating their stories to multiple authorities while receiving little practical assistance. This fragmentation delayed necessary support and deepened a sense of abandonment. Structural barriers such as lacking a fixed address or proper documentation also had direct consequences for their children’s health and wellbeing: “My oldest child has trauma; she is denied psychological treatment for it because we don’t have a stable place of residence.” (Interview 4).

“Mother against Goliath” thus illuminates the lived paradox of moral agency under systemic constraint. This is conveyed by the endurance of women who continuously fought to protect their children while confronting bureaucratic systems that doubted, delayed, and dismissed their needs.

#### Theme 2: Shattered foundations: losing control, connection, and identity in motherhood

The second theme illuminates how the lived meaning of loss, connection, and identity unfolded. The women’s narratives revealed how instability, systemic neglect, and repeated disruptions eroded their sense of self, both as mothers and as women. Motherhood was experienced as emotionally fractured, marked by grief over lost agency, broken attachments, and a persistent struggle to remain present for their children despite overwhelming insecurity. The discrepancy between their intentions and the support they were able to provide their children intensified feelings of guilt, inadequacy, and emotional exhaustion. Motherhood in the context of homelessness meant striving to preserve attachment amid fragmentation, holding on to one’s children emotionally even when separated physically or institutionally.

Systemic failures and chaotic living conditions diminished the women’s sense of agency and brought forth experiences of powerlessness and grief. The mothers expressed sorrow over being separated from their children, which was an emotionally shattering experience. While some acknowledged state intervention might protect the child, having their children involuntarily removed also involved trauma and moral pain. Uncertainty surrounding custody decisions threatened their sense of maternal identity, amplifying anxiety and existential insecurity. Women expressed a deep emotional conflict between wanting to care for their children, and for example, having to vacate housing to avoid active intimate partner violence, or needing to step away to recover from substance use. Consequently, motherhood was experienced as a series of painful moral choices, sacrificing immediate presence to secure long-term safety. To protect their children’s health and safety, some chose to be temporarily absent from their children to focus on rehabilitation. The inability to provide adequate support reinforced self-blame and feelings of failure and shame:

I felt like a failure, limited, that I couldn’t give my children what they needed and what I wanted them to have in their childhood after everything we went through with not having anywhere to live. (Interview 10)

Women experienced that instability, and a lack of secure housing or support networks, were used against them in custody processes. Fear of losing their children led some to remain in abusive relationships, exposing themselves and their children to further harm. Torn family structures and disrupted relationships deepened their sense of grief; personal sacrifices made for their children often jeopardised their own wellbeing. In this process, vulnerability to violence, trauma, and exploitation increased. One mother, who had experienced periods of homelessness and separation from her children, described prioritising their access to support while overlooking her own need for help:

I forgot about myself, I made sure the kids got help but I forgot about myself […] I prostituted myself and self-harmed in every possible way. (Interview 2)

For some women, the loss of control and identity as mothers was most painfully felt in the gradual erasure from their children’s lives. Foster families were sometimes perceived as taking over the maternal role, further deepening the women’s sense of invisibility and displacement. One mother, whose three children had been placed in foster care, described seeing them once a week with the foster carers present. She experienced changes in how her children addressed her as a challenge to her place as their mother and described her efforts to reaffirm that relationship:

You’re a parent even though you’re not really one, and you get to see your kids one day a week […] the family that has my kids tries to persuade them too. […] They put it into their heads and say, ‘We are your mom and dad now.’ […] They’re trying to push me out as a parent. […] One of my sons calls out, ‘Mom’ […] ‘Yes,’ I said, ‘what is it, sweetheart?’ ‘Not you,’ he said to me. […] You fall apart inside, but you don’t show it on the outside […] Then I said […] ‘I am your mother, I am the one who brought you into the world […] You have the privilege of having two parents, two fathers and two mothers, that’s good, but I am still your biological mother and I always will be. (Interview 1)

This narrative captures how losing everyday contact with one’s children becomes an existential loss of recognition. The mothers’ accounts revealed not only grief but also a struggle to preserve identity through words and gestures such as sharing gifts and jokes, thus reclaiming “motherhood” as a moral and embodied truth. In these moments, motherhood was lived as both presence and absence: a love that persisted even when social and relational roles were denied.

Women witnessed emotional and mental distress in their children, not only as a consequence of homelessness but as a reflection of cumulative trauma, exposure to violence, instability, and disrupted attachment. Their children’s distress manifested in different ways, including symptoms of post-traumatic stress, such as sleeping difficulties, fears, and intrusive nightmares. One mother reflected on how her son’s experiences of violence could shape the way he expressed affection:

My son has a crush on a girl who lives here [at the shelter] and he gets so physical with her, so I took him aside and asked, ‘why are you behaving like that with her, she gets really sad’, and then he whispered to me, it’s because I love her. So, they have the horrible conditions they grew up in, they don’t know how to handle their feelings or how to even behave towards people you love. (Interview 8)

Living conditions in protected or temporary housing were experienced as incompatible with children’s needs, reinforcing the mothers’ sense of inadequacy and helplessness. Social isolation due to unstable living conditions further compounded this suffering, as children were unwilling to bring friends home. Limited opportunities for friendship, play, and belonging reflected how homelessness disrupted the ordinary rhythms and rituals of family life. One mother described living with her children for eight months in cramped temporary accommodation without a fully functioning kitchen:

They went to school, but they felt very bad psychologically, they couldn’t bring friends home and I understand that they didn’t want to, and then they didn’t have their own beds or anything, all the furniture was in a storage room like that. (Interview 10)

“Shattered foundations” captures the lived meaning of motherhood in the context of homelessness as a disintegration of safety, belonging, and self. Within conditions of housing instability and separation, the women experienced a continual threat to their maternal identity, suspended between love and loss, presence and absence.

#### Theme 3: Striving to protect and reconnect: resilience in motherhood

The third theme illuminates how women sought to rebuild dignity and connection through everyday acts of care and perseverance. Here, resilience is not merely survival, but a process of reclaiming agency and restoring connection in the aftermath of disruption.

The women’s accounts expressed resilience as both emotional endurance and moral commitment — a determination to care, protect, and remain present for their children amid uncertainty. Despite exhaustion and fragile support systems, mothers described efforts to restore everyday routines and help their children feel secure. After leaving sheltered accommodation and returning to her family network, one mother described:

We got back into routines, going to bed and getting up and so on, but my focus then was on helping the children feel safe again and be able to trust other adults again […] That’s what I’ve worked on most during this period, and today they feel secure in themselves and with other adults. (Interview 9)

The woman’s words convey resilience as moral endurance and protection as an act of love. A commitment to preserve both safety and connection even within conditions of fear and confinement. Thus, reconnection can be understood not as a return to what was, but as a gradual reweaving of trust and belonging through everyday care.

These everyday actions illustrate how women recreated meaning and stability within instability, transforming care into a moral and practical form of endurance. The women’s narratives revealed how care for their children became a moral and emotional anchor, a way of holding on to meaning when life felt fractured. In this way, we interpreted motherhood as both a source of vulnerability and a ground for perseverance. For one mother, who had left an abusive relationship and was living with her children in a domestic violence shelter, resilience and protection were inseparable. She valued the safety the shelter provided, but worried about how the situation affected her children’s schooling, friendships and everyday lives. Uncertainty about how long they would remain there made it difficult to hold on to the hope that their difficulties were temporary:

If the children don’t feel well, I can’t feel well either. At the same time as I know that for safety we have to be here so I am happy that we are here […] so I try to tell myself that this is right, so even though they feel bad, this is still a short time in life, but then when you don’t know how long it will take, then it becomes more difficult. If you knew that in two months it would be over or in three, or six months or something, but when you don’t know, that’s when it will be… But I still see the future brightly, I think it will be good. (Interview 3)

The women strove to protect their children as much as possible and demonstrated agency in seeking resources amid a chaotic life situation. Seeking safety and stability, mothers reached out to multiple sources of support, including health services, social services, and their own networks. They also sought guidance from others whom they perceived as positive parental role models:

I have done the best I could to the best of my ability […] I have taken help from [child and adolescent psychiatry], I have taken help from my drug-free friends, but above all I have gone my own way […] I have looked around at how other, healthy relationships, have done with their children and I have took help. (Interview 2)

Women described suppressing their own emotions and personal pain to protect their children. Emotional detachment became a strategy for survival, a way to maintain focus on their children’s immediate needs and to preserve decision-making capacity in the midst of chaos. While staying in sheltered accommodation with her children, one mother described exhaustion, lack of sleep and difficulty attending to her own needs:

I was like a machine, the only thing… I was downing coffee and energy drinks to keep going, because I was just in survival mode, trying to be there for my children. That was my only focus. I didn’t give a damn about myself as long as they were doing well and we got through the days. (Interview 9)

Over time, as circumstances stabilised, mothers began to rediscover moments of reconnection and relief. Regaining a sense of security enabled them to re-enter the role of being “a real mother” and to rebuild healthy relationships with their children. Having reached safety with her son, one mother described having more energy for parenting and feeling able to set boundaries without fear:

I have finally been able to be a real mother […] he was already involved in so many horrors, so having a normal parent-son relationship now that he’s living without anyone getting mad and that I can set him the right boundaries and lead him, it’s been great to start being able to do that. (Interview 8)

For another woman, secure housing had made it possible to become a mother after years of homelessness. She described:

The last two years I’ve had a secure living situation and that has also meant that you’ve had the opportunity to have children. Because before then it probably wouldn’t have worked. (Interview 7)

A persistent determination to maintain contact with their children was evident, even when physical distance or custody barriers limited interactions. Maintaining regular routines of communication helped mothers sustain emotional closeness and express love across separation. One mother whose children lived with their father described how she expressed her love through regular calls and messages:

I call every day or I write every day and say, ‘Hello, kiss the children on the head, mom loves them today like any other day’. (Interview 12)

Through these narratives, the women described motherhood as an act of moral responsibility and enduring love. Their resilience reflects not only survival but a profound commitment to sustain relational bonds against the odds.

In “Striving to protect and reconnect”, the women described motherhood as a moral and relational practice, a continual act of resilience, protection, and reconnection. Their experiences reveal resilience not only as survival, but as a profound commitment to sustain love and relational bonds against the odds. Motherhood, in this sense, was both a burden and a source of meaning: a way of holding on to dignity, purpose, and hope within instability.

## Discussion

Taken together, the findings elucidate motherhood in the context of homelessness as a process of negotiation, fragmentation, and reconstruction — where maternal love and responsibility persist despite structural barriers and existential loss. These lived experiences reveal how institutional structures and policy practices shape women’s possibilities for wellbeing, safety, and care. Each theme illuminates a particular aspect of this lived meaning: the struggle for agency within systems of control and neglect; the emotional and relational disintegration of identity under structural insecurity; and the moral reconstruction of motherhood through resilience and care.

### Comprehensive understanding

The comprehensive understanding reveals motherhood in the context of homelessness as an existential and moral struggle, shaped by a continuous negotiation between care, loss, and survival within fragmented systems that simultaneously sustain and constrain maternal life. To account for the diversity of maternal experiences in this study, ranging from living in shelters with protected identities due to domestic violence, to separation from children and the pursuit of stability after homelessness, we draw on the concept of *motherhood under structural insecurity* [3]. This concept captures how maternal identities are shaped and constrained by policy logics, stigma, and institutional neglect, particularly when mothers do not conform to normative family models. Situated within the social determinants of health framework [5], the findings show how institutional arrangements powerfully shape the conditions under which women can mother safely and meaningfully. Building on this understanding, the first theme highlights how motherhood unfolds within fragmented and unresponsive welfare systems. These contradictions reflect the “paradox of bureaucracy” [34], whereby systems designed to protect families instead reproduce inequities through rigid rules, mistrust, and conditional support. The findings can also be understood through the concept of structural violence [35], highlighting how institutional arrangements may systematically generate harm. Rice et al. [36] argue that bureaucratic processes can become sources of suffering, requiring repeated demonstration of deservingness. Our study illustrates that welfare systems may intensify challenges associated with homelessness and motherhood, aligned with critiques of neoliberal approaches to motherhood suggesting that support systems place responsibility for caregiving on mothers despite inadequate structural support [37]. Consistent with earlier research [1, 2], mothers must navigate welfare systems that undermine rather than support the very essence of motherhood as a relational and caregiving role, thereby reproducing mistrust and inequality. These institutional dynamics are powerful structural determinants of health. Such bureaucratic indifference operates not merely as inefficiency, but also as an institutional determinant of health that shapes access to care and recognition [5, 38]. Viewed through a health equity lens, these findings underscore the need for welfare systems that recognise and allow for women to sustain the relational, affective and care-based core dimensions of mothering as central to intergenerational health, rather than positioning mothers as economic actors whose success is measured through self-sufficiency.

Evidence from intervention research suggests that barriers hindering access to adequate support can be mitigated through relational and trust-based approaches. The Nurse-Family Partnership provides sustained home-based support to socioeconomically disadvantaged first-time mothers, improving cognitive outcomes and convictions among adolescents. The programme illustrates how sustained professional support can be embedded within services for families experiencing structural disadvantage [39]. Similarly, relational organisational models and community-based participatory approaches emphasise continuity, shared decision-making, and accountability as foundations for ethical and effective support [17, 40]. This underscores that relational ethics is not an abstract ideal but a practical requirement for equitable care.

The second theme illustrates how structural insecurity and institutional separation fragment women’s sense of self, belonging, and maternal identity. Cumulative exposure to homelessness, poverty, and stigma compounded psychosocial distress, supporting prior evidence indicating that such suffering is socially produced rather than solely produced on an individual level [1, 41]. For some women, separation from their children was experienced as both a necessary act of protection and a profound rupture of identity. These findings expose a central ethical tension in child protection systems as safeguards for the safety of children while dismantling the relationships that sustain maternal and child wellbeing.

Research suggests that this ethical tension need not be resolved through severance. Preserving safe and meaningful contact between mothers and children in out-of-home care can support relational continuity, maternal identity, and children’s emotional wellbeing, provided that their contact is responsive to the children’s needs [42]. Integrated care and substance-use treatment responsive to relational dynamics may reduce child removal and enhance maternal engagement in care [43]. At the same time, evidence on child development highlights that stability and continuity of care, rather than biological ties alone, are central to children’s wellbeing [44, 45]. Our findings emphasise the emotional strains associated with family disruptions and call attention to the importance of carefully involving mothers when planning how to ensure the safety of their children.

The third theme reveals motherhood as a moral and health-promoting practice. Despite trauma, stigma, and loss, women enacted forms of care that sustained connection, dignity, and meaning beyond mere survival. Maintaining daily and social routines such as sharing meals functioned as moral practices through which women exercised agency under constraint. Consistent with process-oriented models of resilience, these acts were shaped through the interplay of personal endurance, relational bonds, and institutional conditions that both facilitated and hindered processes of social and emotional recovery within broader systems of adaptation [6, 12].

Emotional regulation was central to motherhood as a practice to foster moral responsibility, health and wellbeing. The participants suppressed their own distress to remain emotionally available to their children, a strategy aligned with research on parenting stress under adversity [46]. In line with Medina’s [7] account of moral agency under oppression, such everyday acts of care can be understood as forms of resistance that affirm dignity and relational belonging. In this sense, resilience appears to involve relational and moral practices. Resilience can therefore be seen as an ongoing effort to restore coherence, humanity, and health within the structural insecurity of motherhood in the context of homelessness. Through an inclusion health approach [15, 16], the findings call for health and welfare systems that recognise relational caregiving as central to recovery, equity and inclusion.

### Strengths and limitations

There are ethical complexities related to participatory research with people in vulnerable situations, particularly when academic researchers and experts with lived experience collaborate as co-researchers. Careful ethical reflection was therefore needed throughout the research cycle, extending beyond traditional researcher–participant relationships. For example, peer interviewing conducted by women with lived experience of homelessness was not possible due to confidentiality and safety requirements in protected housing situations, illustrating both the ethical sensitivity and institutional constraints of participatory research in these settings.

In line with Ricoeur’s [27] view that interpretation is inherently plural, Lindseth and Norberg’s phenomenological–hermeneutic lens offered a structured yet interpretive analytic framework suitable for this study [18, 19]. We argue that the reflexive and collaborative design in this study strengthened its ethical and interpretive integrity, as we strived to keep women’s lived experiences central to the knowledge co-production. Careful attention was given to the representation of participants’ voices in translation and writing. Decisions regarding wording were discussed collaboratively, and participants’ original phrasing was retained where possible to preserve lived meaning. Guided by reflections from a co-researcher with lived experience of motherhood in homelessness, the team prioritised authenticity over linguistic sanitisation, recognising that modifying language risked imposing a “double shame” on participants.

This exploratory and interpretative qualitative study aimed for analytic depth providing insights into a complex and context-sensitive phenomenon. Participants were recruited for their capacity to provide information-rich and diverse perspectives aligned with the study aim, striving for a variation in experiences within the sample. We acknowledge that the twelve women in our sample each carries her own unique lived experience, background and characteristics. In line with the concept of information power [47], we assessed that the sample provided sufficient depth, specificity and variation to address the study aim. Although efforts to recruit participants continued for more than six months, recruitment was concluded following discussions within the research team, which found that the material offered a sufficiently rich basis for interpreting and understanding the lived experiences of motherhood in the context of homelessness. In addition, we sought to balance recruitment efforts with consideration for participants’ circumstances and to avoid imposing undue burden on women facing challenging life situations. Nevertheless, there are limitations regarding the transferability of the findings, as only participants able to communicate in Swedish were included in the interviews. Affective and relational dimensions of motherhood, particularly under conditions of structural insecurity, may resist articulation or remain partially unspoken. This entails that any account of mothers’ lived experience is necessarily partial, and therefore, underscores the need for continued inquiry that attends to mothers’ expressions beyond verbal utterances alone.

### Concluding interpretation

Motherhood in the context of homelessness is a deeply relational and moral experience shaped by ongoing tensions between care and constraint. Women’s efforts to mother are negotiated within fragmented systems that simultaneously sustain and undermine them, including welfare bureaucracies demanding compliance, institutional logics fragmenting care, and socioeconomic hierarchies defining who counts as a “good” mother. Viewed through the lens of the social determinants of health [5], institutional arrangements operate as powerful determinants of health and wellbeing, generating both psychosocial suffering and, paradoxically, opportunities for resilience.

Distress and agency should not be viewed as opposites, but as intertwined responses to structural insecurity. The same conditions that erode safety and belonging may also prompt women to be creative and display moral acts of care. The findings further point to epistemic injustice [7], illustrating that women’s embodied and relational knowledge can be overlooked or devalued within institutional and policy contexts. These lived experiences challenge dominant assumptions about credibility and expertise in health and social support services. From a feminist ethics of care perspective, this marginalisation reflects how gendered ways of knowing are systematically made less legitimate within bureaucratic systems that privilege self-sufficiency over care and affective relationality. In this sense, motherhood under structural insecurity [3] becomes both an existential struggle and a political practice to assert dignity, knowledge and meaning within systems that often fail to recognise these.

There is a need for policy and practice frameworks that address institutional determinants alongside social and material ones. Mothers need to be supported not as passive recipients of care, but as moral agents embedded in complex social realities. By attending to the lived experiences of the intersections of motherhood, structural insecurity and institutional neglect, this study underscores the importance of advancements in health equity as well as equity in care, as two intertwined and inseparable concepts. Advancing health equity for mothers experiencing homelessness requires welfare and health systems that prioritise relational trust, continuity of support and the recognition of lived experience as legitimate knowledge.

## Supplementary Information

Below is the link to the electronic supplementary material.

Supplementary Material 1

## Data Availability

The datasets generated and/or analysed during the current study are not publicly available owing to ethical considerations, including the sensitive nature of the research topic and the living situations of the participants.

## Abbreviations

CBPR: Community-based participatory research
COREQ: Consolidated Criteria for Reporting Qualitative Research
CRC: United Nations’ Convention on the Rights of the Child
ETHOS: European Typology of Homelessness and Housing Exclusion
NIHR: National Institute for Health and Care Research
WAB: Women’s Advisory Board for Inclusion Health
